# Percutaneous Ethanol Sclerotherapy of Symptomatic Nodules Is Effective and Safe in Pregnant Women: A Study of 13 Patients with an Average Follow-Up of 6.8 Years

**DOI:** 10.1155/2015/765950

**Published:** 2015-11-30

**Authors:** Tamas Solymosi, Zsolt Melczer, Istvan Szabolcs, Endre V. Nagy, Miklos Goth

**Affiliations:** ^1^Thyroid Outpatient Department, Bugat Hospital, 6 Fenyves Street, Gyongyos, Matrafured 3232, Hungary; ^2^Department of Obstetrics and Gynecology, Semmelweis University, Üllői Street 78/A, Budapest 1082, Hungary; ^3^Division of Endocrinology, Medical Centre of Hungarian Defense Forces, 44 Robert Karoly Avenue, Budapest 1134, Hungary; ^4^Division of Endocrinology, Department of Medicine, Faculty of Medicine, University of Debrecen, Nagyerdei Square 94, Debrecen 4032, Hungary

## Abstract

*Background.* Because of the increased risk of surgery, thyroid nodules causing compression signs and/or hyperthyroidism are concerning during pregnancy.* Patients and Methods.* Six patients with nontoxic cystic, four with nontoxic solid, and three with overt hyperthyroidism caused by toxic nodules were treated with percutaneous ethanol injection therapy (PEI). An average of 0.68 mL ethanol per 1 mL nodule volume was administered. Mean number of PEI treatments for patients was 2.9. Success was defined as the shrinkage of the nodule by more than 50% of the pretreatment volume (V0) and the normalization of TSH and FT4 levels. The average V0 was 15.3 mL. Short-term success was measured prior to labor, whereas long-term success was determined during the final follow-up (an average of 6.8 years).* Results.* The pressure symptoms decreased in all but one patient after PEI and did not worsen until delivery. The PEI was successful in 11 (85%) and 7 (54%) patients at short-term and long-term follow-up, respectively. Three patients underwent repeat PEI which was successful in 2 patients.* Conclusions.* PEI is a safe tool and seems to have good short-term results in treating selected symptomatic pregnant patients. Long-term success may require repeat PEI.

## 1. Introduction

Although the evaluation of a pregnant patient with a thyroid nodule does not differ from other patients except in the use of scintigraphy [1–3], the management of TN during pregnancy is discussed only in the context of malignancy. The only suggestion in the context of the management and therapy of TN during pregnancy is the statement that levothyroxine is not recommended during pregnancy [2]. The ATA guidelines published in 2011 recommend surgery in the case of rapid nodule growth and/or if severe compressive symptoms develop [4].

Data indicate that pregnancy is associated with an increase in the size of preexisting nodules and with the appearance of newly developed thyroid nodules, possibly because of the negative iodine balance that frequently occurs during pregnancy [5]. Depending on iodine intake, thyroid nodules are present in 15.4 to 30.1% of pregnant women [6, 7].

Therapy (i.e., surgery of benign nodules) in such women must be postponed after delivery if possible. Nevertheless, TN causing pressure symptoms and/or hyperthyroidism raises concern about appropriate therapy because surgery and general anesthesia pose risks in pregnant women [8], and the use of thyrostatic agents is not without risk. Although percutaneous ethanol injection therapy (PEI) is a useful alternative to surgery in recurrent thyroid cysts and may have a limited role in the case of solid TN, to the best of our knowledge, there are only two publications focusing on [9] or mentioning [10] PEI in the event of pregnancy. Based on this information, we decided to analyze our experiences in this field.

## 2. Patients and Methods

Between June 1996 and December 2013, 267 pregnant patients with thyroid nodules were evaluated in our thyroid outpatient clinic. We offered PEI therapy to 10 patients with significant pressure symptoms and to 3 patients with toxic nodules with an FT4 level exceeding the upper normal value by at least 30%. Eight women accepted our invitation. Five other patients were evaluated in other thyroid departments and were referred to us for PEI therapy. Thirteen pregnant patients with thyroid nodules were treated with PEI. TSH, FT4, anti-thyroid peroxidase antibody (aTPO), anti-human thyroglobulin antibody (ahTG), and ultrasonography were performed in all patients prior to PEI. Scintigraphy was performed in 3 patients prior to their pregnancies. The volume of the nodules was calculated as described by Brunn et al. [11]. The clinical data are presented in Table 1.

The indications for PEI were the following: recurrent cyst causing pressure symptoms in 6 patients and large nontoxic solid nodule causing symptoms in 4 patients. Three patients had toxic nodules, one of which caused symptoms, a second of which increased by more than 100% in volume from the 5th to the 15th gestational week, and the third occurring in a woman who was treated with propylthiouracil and exhibited a neutrophil count of 1,300 (normal value 4,000–10,000) 2 weeks after initiating the thyrostatic drug. Prior to the PEI treatment, malignancy was ruled out in all patients by fine needle aspiration cytology. The aTPO and ahTG (Brahms, Berlin, normal value <100 U/mL) levels were normal in all patients, as were the TSAb levels in the 3 patients exhibiting hyperthyroidism. We asked the patients about the presence of their compression signs. The degree of pressure symptoms was defined as described in Tables 1 and 2.

In the case of nontoxic nodules, PEI was performed between the 16th and the 22nd gestational week of pregnancy. Patients with toxic nodules were treated as soon as possible after the 13th gestational week of pregnancy.

Sterile 95% ethanol was injected under US control via a 23-G needle without anesthesia or pharmacological sedation. Depending on the nodule size, the ethanol dose injected in each session, which was administered slowly over 2 minutes, varied from 1 to 6 mL. In the case of cystic nodules, 90% of the cystic volume was aspirated prior to PEI, and an ethanol volume equivalent to 20% to 33% of the volume of the aspirated fluid was injected. In the case of toxic and nontoxic solid nodules, an ethanol volume representing between 20 and 30% of the initial volume of the nodule was administered. PEI sessions were performed every 7 days except in the case of toxic nodules, which were treated every 3 days (see Table 1).

Each patient received an average total dose of 0.68 mL ethanol (range 0.43–0.91 mL) per milliliter nodular volume. The ethanol was injected in an average of 2.92 (range 2–4) outpatient sessions.

During PEI treatment, 2 patients were treated with propylthiouracil, which was stopped on the day of the last PEI session. Thyrostatic therapy was stopped in the 3rd patient with a low neutrophil count before PEI therapy.

The follow-up protocol included physical examination; US; TSH, FT4, aTPO, and ahTG measurements; and the determination of degree of compression signs. The follow-up was performed every 4 weeks during pregnancy and annually thereafter. In the case of toxic nodules, the first TSH and FT4 measurement was performed 2 weeks after the last PEI.

For nontoxic nodules, the outcome of the PEI treatment was determined by US examination. Success was defined as the ≥50% shrinkage of the nodule relative to the pretreatment volume every 8 weeks and during every follow-up occasion. For toxic nodules, complete success required the fulfillment of one additional condition: normal TSH and FT4 levels 4 weeks after PEI and later in the course without thyrostatic therapy. We determined the short-term (until delivery) and long-term (until the final follow-up examination) success of PEI.

For statistical analysis, a repeated measures ANOVA test was used to compare changes in the volume of the nodules and in TSH levels, and the Friedman ANOVA test was applied to compare changes in the degree of compression signs.

## 3. Results

### 3.1. Side Effects

In 7 of 18 sessions and in 17 of 20 sessions, patients reported pain during the ethanol injection in the cystic and noncystic nodule groups, respectively. The pain radiated to the jaw and the teeth. With the exception of two sessions, the pain decreased significantly over 2 to 3 minutes. Pain stopped between 1 and 24 hours after injection in 2 patients.

Mild neck discomfort was reported in 28 of 38 sessions and resolved within 24, 48, 96, and 124 hours after 11, 10, 6, and 1 PEI session(s), respectively.

The voice of Patient 6 weakened after the last PEI. This effect lasted for 36 hours, and her voice returned spontaneously without any treatment.

### 3.2. Change in the Degree of Pressure Symptoms

Eleven patients suffered from various degrees of pressure symptoms before PEI (see Table 2). In all but one patient, the degree of compression decreased 4 weeks after the last PEI and remained stable until delivery. The degrees of pressure symptoms were 1.84 ± 1.11, 0.31 ± 0.48, and 0.62 ± 0.968 (mean ± SD) before the first PEI, at the last visit before delivery, and at the final visit, respectively. The differences between the first and second visit and between the first and final visit were significant (*p* < 0.005 and *p* < 0.011, resp.).

### 3.3. Short-Term Results (prior to Delivery)

All but two women delivered a healthy child between the 38th and 41st gestational week. One woman (Patient 5) developed late-onset preeclampsia (with hypertension and proteinuria) at the 35th gestational week and delivered a healthy girl weighing 2730 g at the 36th gestational week. This patient was treated for a recurrent cystic nodule between the 18th and 21st gestational week and was euthyroid at the onset of preeclampsia. Patient 13 experienced a premature delivery at 33 weeks. She was treated for a toxic nodule between the 13th and 14th gestational week and became euthyroid 2 weeks later, remaining euthyroid until the final visit 5 years later. She delivered a baby weighing 2250 g at birth who was discharged in a healthy condition 10 days after delivery. Ten women experienced vaginal deliveries, and 3 underwent caesarean deliveries. The reasons for caesarean were unrelated to thyroid disease.

For details of volume and biochemical data, see Table 2. In all but one patient, the volume of the nodule at the last predelivery visit was less than 50% of V0. In Patient 2, who had a cystic nodule, the predelivery volume was 61.4% of V0. The mean V0 was 15.3 ± 8.0 mL, whereas the mean volume at the last predelivery visit was 5.68 ± 4.01 mL. The difference was statistically significant (*p* < 0.0001).

Thyrostatic drugs were stopped on the day of the last PEI in two patients and before the first PEI in the case of a third patient with a low neutrophil count. The FT4 level normalized and remained stable in all patients after the first follow-up visit and during the pregnancy. Two of the 3 patients with toxic nodules remained euthyroid (i.e., the TSH became and remained normal during their pregnancy), whereas the 3rd patient (Patient 11) with the largest nodule exhibited subclinical hyperthyroidism later in the course of pregnancy with a TSH in the range of 0.01–0.07 mIU/L and with an FT4 in the range of 12.1–15.0 pM/L. None of the patients was treated with thyrostatic drug after the last PEI session.

ATPO and ahTG remained normal during pregnancy in all patients.

Short-term success was reached in 11 of 13 patients; in the remaining 2 women, compression signs ceased, and the patients required neither thyrostatic therapy nor surgery until labor.

### 3.4. Long-Term Results

Thyroid status gradually worsened in Patient 11, who exhibited subclinical hyperthyroidism at the last predelivery visit. Two years later, Patient 11 underwent radioiodine therapy. The other 2 patients with toxic nodules remained euthyroid, and the volume of their nodules did not exceed 50% of V0 5 and 11 years after PEI, respectively.

In 3 of the 4 patients with nontoxic solid nodules, the volume of the nodules exceeded 50% of V0, 7, 8, and 10 years after PEI, respectively. It is worth noting that, one year earlier, the volume of these nodules was less than 50% of V0. The volume of the nodule of the fourth patient remained less than 50% of V0 3 years after her delivery.

PEI was unsuccessful during the short-term in Patient 2. Four of the remaining five patients with cystic nodules exhibited a nodule volume less than 50% of V0 at the last visit, 2, 6, 9, and 11 years after PEI, respectively.

The volume of the cystic nodule in Patient 5 was 25.0% of V0 2 years after PEI, but, thereafter, she noticed a sudden increase in size with a 98.3% volume of V0 29 months after PEI.

The mean volume at the final visit was significantly lower compared with V0 (7.0 ± 5.1 mL and 15.3 ± 8.0 mL, resp., *p* < 0.0001). The volume at the final visit was not significantly greater than that at the last predelivery visit (*p* = 0.89).

ATPO and ahTG were normal in all patients at each follow-up examination except for Patient 3, who was treated for a cystic nodule. aTPO became elevated one year after delivery (128 U/mL) and fluctuated in the range of 61 and 143 U/mL during the following 8 years.

Long-term success was reached in 7 of 13 patients with one series of PEI.

### 3.5. The Fate of Patients Whose PEI Was Unsuccessful

For details, see Table 3. As mentioned earlier, Patient 11, who had a toxic nodule, underwent radioiodine therapy 2 years after PEI.

One series of PEI was unsuccessful in 3 of 4 patients with nontoxic solid nodules. Patient 10 underwent surgery. Patient 7 repeated PEI therapy 7 years after the first PEI. Her nodule was less than 37.8% of V0 2 years after the second PEI. The nodule in Patient 8 was 68.5% of V0 10 years after PEI but did not require surgery until recently.

PEI was unsuccessful in 2 of 6 patients with cystic nodules. The volume of the nodule of Patient 2 was 69.1% of V0 18 months after the first PEI, when she received 3 more PEI sessions. Her nodule was 12.0% of V0 6 years after the second session of PEI. We administered 3 more PEI sessions 29 months after the first PEI in Patient 5 with only transitory success for another 2 years. Patient 5 subsequently underwent lobectomy.

One series of PEI was unsuccessful in 6 patients. Three of these patients underwent a repeat PEI, which was successful in 2 but was unsuccessful in 1 patient.

## 4. Discussion

PEI therapy is an accepted method for the treatment of recurrent thyroid cysts; however, it is not currently recommended for solid nodules because of weak long-term results [1, 12]. Nevertheless, PEI may be a useful alternative to surgery in the case of solid nodules under special circumstances, such as recurrent nodules [13], iodine-induced hyperthyroidism [14], particular cases of lymph node metastasis of papillary carcinoma [15], large nodules in which the main goal is to gain time (i.e., patients with poor life expectancy) [16], patients with special professions, and pregnant patients [9, 14].

Thyrostatic therapy is relatively safe during pregnancy and the surgical therapy of most pregnant patients can be postponed after delivery. Nevertheless, in a small proportion of pregnant patients with nodular goiter, mainly in those presenting with significant compression or having significant side effects of medical therapy, there is a need for alternative therapy during pregnancy.

There are few publications in the field of PEI therapy concerning pregnant women. Cortelazzi et al. published a case report of a patient with a toxic nodule and stated that PEI may be a safe therapy during pregnancy [9]. We published our experience in 1999 with 2 pregnant patients with cystic nodules and found that PEI therapy was safe and efficient [10].

With respect to the short-term goals, the pressure symptoms stopped or decreased in all but 1 of the 11 patients presenting with the complaint before PEI, whereas FT4 became and remained normal without thyrostatic therapy until delivery in all toxic patients. Statistically, PEI was not fully successful in 2 patients. The volume of the nodule remained greater than 50% of V0 at the last predelivery visit (61.4%) in one woman. Nevertheless, her serious pretherapy compression sign completely ceased after PEI and did not recur later in the course. In one of the 3 toxic patients, the TSH remained suppressed at the time of delivery, but the FT4 level normalized and stabilized after PEI and until delivery without thyrostatic therapy.

With respect to the long-term results (with an average follow-up of 6.8 years), PEI was successful in 7 of 13 patients. Compared with PEI in nonpregnant patients, this result represents a lower response rate. Our most important goal was to ensure optimal thyroid conditions until delivery with minimal risk of any deleterious effects of PEI; therefore, we administered only an average of 2/3 of the recommended 1 mL ethanol per milliliter nodular volume. We think that this amount explains the poor long-term results. Three of 6 patients with unsuccessful PEI required surgery or radioiodine therapy, and, in two patients, a repeated PEI was successful 7 and 2 years after the second therapy, respectively. The volume of the nodule in the sixth patient was 68.5% of V0 10 years after PEI but did not require surgery until recently.

An aTPO positivity may develop after PEI [17, 18]. This effect would have been a potential drawback during pregnancy. No aTPO or ahTG positivity developed during pregnancy in our patients, and, later in the course, we observed aTPO elevation in one patient one year after delivery.

The success of PEI decreased significantly with the length of follow-up time. We determined PEI to be unsuccessful in 3 of 6 patients after more than 7 years of follow-up.

The present study demonstrates that PEI therapy may be a useful alternative in pregnant patients with nodules causing compression signs. No serious side effects occurred. All but 2 women delivered on time and had no complications during their pregnancy and labor. Neither the premature delivery of one woman at 33 weeks nor the late-onset preeclampsia in another woman was related to thyroid disease or to PEI therapy. While PEI may have a role in the therapy of large cystic nodules causing compression signs, in solid nodules laser or radiofrequency percutaneous thermoablation seems to be more efficacious and less painful than PEI.

## Figures and Tables

**Table 1:** Pretreatment clinical data on patients.

	Age (ys)	Gestational week at 1st session	Degree of compression^1^	Size of nodule (mL)	Aspirated cystic fluid (mL)	Number of sessions	TSH (mIU/L)	FT4 (pM/L)
*Type of nodule*								
Nontoxic, cystic	22	16	2	17.9	9	3	1.01	13.9
Nontoxic, cystic	25	19	3	24.9	14	3	0.87	14.6
Nontoxic, cystic	29	16	3	30.6	24	3	3.01	12.7
Nontoxic, cystic	34	17	2	8.6	6.5	2	0.33	15.1
Nontoxic, cystic	39	18	3	11.9	5.7	3	2.39	18.4
Nontoxic, cystic	43	19	3	24.7	0.5	4	1.52	11.3
Nontoxic, solid	30	20	2	10.1	0	3	2.76	15.4
Nontoxic, solid	32	16	1	8.9	0	2	0.97	13.7
Nontoxic, solid	35	17	3	13.5	2.1	3	1.62	17.0
Nontoxic, solid	37	17	1	21.9	0	4	2.11	20.2
Toxic	20	16	1	13.8	2.9	3	0	43.9
Toxic	31	15	0	8.04	2.8	3	0^2^	29.8
Toxic	39	13	0	4.32	0.7	2	2.91^2^	8.05

Mean ± SD	32 6,86		1.84 1.14	15.3 8.0		2.92 0.64	1.66^3^ 0.89	15.2^3^ 2.67

^1^Degree of compression: 0: none; 1: occasionally in supine position; 2: continuous in supine position; 3: even in upstanding position.

^2^On propylthiouracil therapy.

^3^The mean TSH and FT4 values were calculated only in nontoxic nodules.

**Table 2:** The effect of PEI therapy on pressure symptom, nodule volume, and TSH-level^1^.

Number	Type of nodule	Age (ys)	Follow-up (ys)	Total amount of ethanol, mL (% of pretreatment volume)	Degree of pressure symptoms^2^			Size of nodule, mL (% of pretreatment volume)			TSH (mIU/L)			Final result of one series of PEI
					Before therapy	On last visit before delivery	On final visit	Before therapy	On last visit before delivery	On final visit	Before therapy	On last visit before delivery	On final visit	
1.	Nontoxic, cystic	22	12	10.8 (60.3)	2	1	0	17.9	3 (16.7)	1.8 (10.1)	1.01	1.20	1.04	Success

2.	Nontoxic, cystic	25	2	10.7 (43.0)	3	0	0	24.9	15.3 (61.4)	17.2 (69.1)	0.87	1.11	2.91	No success

3.	Nontoxic, cystic	29	9	21.5 (70.3)	3	0	1	30.6	8.1 (26.5)	6.9 (22.5)	3.01	2.58	2.73	Success

4.	Nontoxic, cystic	34	16	4.6 (53.5)	2	0	0	8.6	2.2 (25.6)	0.7 (8.1)	0.33	0.76	0.69	Success

5.	Nontoxic, cystic	39	2	6.8 (57.1)	3	1	3	11.9	2.6 (21.8)	11.7 (98.3)	2.39	1.99	1.54	No success

6.	Nontoxic, cystic	43	2	18.4 (74.5)	3	1	1	24.7	9.0 (36.4)	7.2 (29.1)	1.52	1.43	1.47	Success

7.	Nontoxic, solid	30	7	8.4 (83.2)	2	0	1	10.1	4.7 (46.5)	7.9 (78.2)	2.76	2.26	2.31	No success

8.	Nontoxic, solid	32	10	5.5 (61.8)	1	0	0	8.9	3.6 (40.4)	6.1 (68.5)	0.97	1.34	1.13	No success

9.	Nontoxic, solid	35	3	9.7 (71.9)	3	0	0	13.5	5.1 (37.8)	5.1 (37.8)	1.62	1.16	1.25	Success

10.	Nontoxic, solid	37	8	15.5 (70.8)	1	1	2	21.9	10.4 (47.5)	15.5 (70.8)	2.11	2.02	2.79	No success

11.	Toxic	20	2	8.9 (64.5)	1	0	0	13.8	5.7 (41.3)	6.4 (46.4)	0	0.03	0.001	No success

12.	Toxic	31	11	6.2 (77.5)	0	0	0	8.0	3.1 (38.8)	3.9 (48.8)	0^3^	0.91	1.10	Success

13.	Toxic	39	5	3.9 (90.7)	0	0	0	4.3	1.2 (27.9)	0.9 (20.9)	2.91^3^	0.45	0.8	Success

	Mean ± SD	32 6,86	6.84 4.61	10.1 (67.6) 5.40 (12.8)	1.84 1.14	0.31 0.48	0.62 0.96	15.3 8.0	5.68 (36.0) 4.01 (12.2)	7.02 (46.8) 5.14 (28.4)	1.66^4^ 0.89	1.59^4^ 0.59	1.79^4^ 0.82	

^1^The results of one series of PEI are given in this table even in those patients who get a 2nd series of PEI.

^2^Degree of pressure symptom: 0: none; 1: occasionally in supine position; 2: continuous in supine position; 3: even in upstanding position.

^3^On propylthiouracil therapy.

^4^The mean TSH-levels were calculated only in the case of nontoxic nodules.

**Table 3:** The fate of patients with unsuccessful PEI therapy.

Number	Type of nodule	Age (ys)	Size of the nodule before first PEI (V0), mL	Time elapsed until 2nd therapy (ys)	Size of nodule before 2nd therapy, mL (% of V0)	Type of 2nd therapy	Last visit after the 2nd therapy (ys)	Size of nodule on the last visit (% of V0)	Final result
2.	Nontoxic, cystic	25	24.9	2	69.1	Repeated PEI	6	12.0	Success
5.	Nontoxic, cystic	39	11.9	2	98.3	Repeated PEI	2	81.6	Surgery
7.	Nontoxic, solid	30	10.1	7	78.2	Repeated PEI	2	37.8	Success
8.	Nontoxic, solid^1^	32	8.9	10	68.5	No therapy	—		
10.	Nontoxic, solid	37	21.9	8	70.8	Surgery	—		
11.	Toxic	20	13.8	2	46.4	I-131 therapy	—		

^1^Although one PEI-series was unsuccessful in Patient 8, she requires no second therapy because her nodule causes no problem.
